# Not snowflakes: Gen Z reflections on rising youth mental ill-health

**DOI:** 10.1016/j.lanwpc.2026.101926

**Published:** 2026-07-23

**Authors:** Georgie Berry

**Affiliations:** The Matilda Centre for Research in Mental Health in Substance Use, The University of Sydney, Camperdown, New South Wales, Australia

## Introduction

Public commentary often portrays young people as a fragile “snowflake generation,” supposedly less resilient than our elders. These claims collapse under empirical scrutiny: across countries in the Western Pacific region, data show rising proportions of young people meeting criteria for mental disorders.^1^

This Viewpoint accompanies the companion article by Birrell, Slade and colleagues, which reports that between 2007 and 2022 the proportion of young Australians meeting criteria for a mental disorder rose from 23.4% to 36.2%, with suicidal thoughts and behaviours more than doubling.^2^ The question is therefore not whether youth distress is increasing, but how those increases should be interpreted.

To inform interpretation of those findings, the study team engaged the Matilda Centre Youth Advisory Board (YAB), a diverse national group of young people with lived and living experience of mental health and substance use through their own experiences, families, and communities.^3^ The YAB was consulted to explore potential drivers of increasing rates of distress and the social narratives surrounding youth mental health, including the “snowflake generation” stereotype.

Rejecting deficit framings, insights from the YAB highlight how economic insecurity, precarious work and housing, the lingering productivity and educational impacts of COVID-19, and the double-edged influence of digital media that may intensify youth distress. The data demand we stop pathologising a generation reacting rationally to irrational conditions. We refuse to frame a generation’s pain as personal failure.

Together with my YAB colleagues, I counter the “snowflake generation” characterisation by arguing that rising youth distress may reflect a growing mismatch between the expectations placed on young people and the increasingly unstable social and economic systems they must navigate.

By centring youth voices, this Viewpoint reflects the principle of “nothing about us without us”: as a priority population, young people must be embedded in research and policy settings, with the goal to enable leadership in areas that directly impact our lives.^3^

**Positionality statement**

I write in a dual role as both a youth mental health researcher and a member of the Matilda Centre Youth Advisory Board.

This commentary synthesises three sources:

1. Findings from the companion national survey analysis
2. Insights generated through Youth Advisory Board (YAB) consultation
3. Interpretation drawing on youth lived experience and relevant literature.

All YAB contributions were anonymised in meeting minutes, and members were informed through project materials that their feedback could be used in publications, with post-meeting opt-out procedures confirming consent for inclusion of de-identified quotations.

## Findings and reflections

### Pressures reshaping youth mental health

When asked, *‘What do you think are some of the common misunderstandings when increasing rates of mental ill health in youth get discussed?’,* one YAB member responded: ‘That mental ill health is attributed to one particular issue, for example social media, COVID-19, climate, war, or the cost of living.’

That line spoke for most of the YAB, with no one naming a singular cause for rising rates of distress. Youth distress is not monolithic; it is cumulative. Members described pressures across work, relationships, finances, climate anxiety, health care access, stigma, and education systems that intersect and accumulate. The issue was not what caused initial distress, but how difficult it has become to live with it.

The companion paper found that comorbidity and functional impairment among young people have both increased.^2^ Members noted this pattern was unsurprising. The YAB outlined that our current support systems aim to treat symptoms, but neglect to appropriately manage the conditions that create them. For many, therapy and medication help only enough to continue functioning within the unstable systems causing their distress in the first place.

The “why” resides in the mismatch between instability and expectation. Young people are asked to plan for a future that no longer exists. Stable work, secure housing, and predictable life stages have been eroded, but socially and institutionally, our successes are still measured by these outdated benchmarks and milestones.^4^ This has lit and stoked the flames of chronic anticipatory stress, not just anxiety about the present, but about whether the social contract still holds and where young people fit in our society. *We are told to plan our futures, while the scaffolding rots.*

Current policy settings remain misaligned with youth realities. In Australia, responsibility for housing, health, and education is divided across state and federal levels, a fragmentation that deepens each year and leaves widening gaps in policy and funding. Youth policy sits within the education portfolio; there is no dedicated federal ministerial portfolio for children and young people.^5^ Without a dedicated portfolio, responsibility for youth wellbeing is dispersed across departments, limiting national accountability and coordination of prevention, policy coherence across sectors, and long-term strategy. Individualised mental health-related treatment approaches, without systems reform, will lead to a continued rise in distress.

### A cultural win, but an unequal one

Reduced stigma surrounding mental illness is one of the most visible public health victories of my generation. Gen Z speaks about anxiety, trauma, and burnout with fluency that was reserved for clinics, hospitals, and research centres. Most of us learned the language of mental health and wellbeing through school and our peers, rather than professionals. That shared vocabulary has been powerful enough to demand response. This increased willingness to recognise distress and seek help may reflect not fragility but growing mental health literacy and agency among young people.

Progress follows a pattern; it reaches those near the centre first. In Australia, the decline in stigma has mostly benefited young people who are urban, economically secure, and white. The more someone deviates from the dominant script, the more their distress is questioned and their access to support systems curtailed. First Nations, culturally and linguistically diverse, disabled, rural and regional, and LGBTQIA + youth remain at a disadvantage to their peers.^6^, ^7^, ^8^, ^9^ These are groups whose experience of stigma collides and intersects with identity-based discrimination and may produce both delayed diagnosis and poorer prognosis in managing mental ill health. Similar disparities are evident across the Western Pacific, where urban privilege shapes access to health services, knowledge, and infrastructure.^10^

Our systems still display bias in deciding whose pain is legitimate, whose language is credible, and which stories are considered worthy of attention, publication, and resourcing. As another YAB member noted: “The prominence of mental illness in communities seems to be a reflection of society’s ill health.”

### Recognition without repair

Help-seeking by young people has risen sharply over the past decade, yet service access and recovery rates have not kept pace.^2^^,^^11^ Systems still expect young people to navigate adult-centred pathways that are complex, fragmented, and often discouraging. Awareness has outpaced prevention and accessibility.^12^

This is *recognition without repair*: a mental-health landscape that celebrates awareness and education but delivers little structural change. Services remain culturally inconsistent, geographically inaccessible, and rarely designed to include families or peer networks.^12^ Both prevention and postvention remain chronically underfunded.

Support must be linguistically accessible, community-informed, and embedded in networks of care, or it will continue to fail those who need it most. Awareness campaigns are not enough. Prevention must start earlier, reach further, and be built for the real-world conditions shaping youth mental health, not just the symptoms reported (Table 1).

**Table 1:** Policy and research priorities for youth mental health systems.

**Research priorities**	**Policy priorities**
1. Mapping youth mental health care pathways Identify where young people disengage from care across primary, community, and specialist services. Research should prioritise system redesign that improves continuity, reduces navigation burden, and aligns services with youth life stages. 2. Measuring equity in mental health access and outcomes Develop national metrics that track disparities in service availability, utilisation, and recovery outcomes across youth populations, including First Nations, culturally and linguistically diverse, rural and regional, disabled, and LGBTQIA + young people. 3. Prevention trials addressing structural drivers of distress Intervention research should move upstream, testing policies and programmes that address housing insecurity, precarious employment, and education environments as determinants of youth mental health. 4. Understanding digital environments as mental health exposures Algorithm driven digital platforms may shape social comparison, information flows, and emotional contagion.^13^ Research could treat digital environments as population level exposures rather than individual behavioural risks. 5. Youth led implementation and governance research Young people should be embedded as partners in research design, implementation, and evaluation. Youth governance models must be studied to determine how lived experience can meaningfully influence mental health systems.	1. National leadership for youth wellbeing Australia lacks a standalone federal minister for children and young people.^5^ A dedicated portfolio would enable national coordination across health, housing, education, and employment policy, and establish clear accountability for youth wellbeing. 2. Youth centred mental health care pathways Current systems require young people to navigate fragmented services across education, primary care, and specialist mental health.^14^ National reform should prioritise simplified entry points, continuity of care, and youth designed services. 3. Rebalancing investment toward prevention Despite rising prevalence of youth mental distress, funding remains heavily weighted toward crisis response and treatment.^15^ Prevention and early intervention must be resourced at scale and embedded across education, community, and social policy. 4. Equity as a core system design principle Structural inequities continue to shape recognition, diagnosis, and access to care. Services must be culturally safe, geographically accessible, and co designed with communities most affected by mental health disparities.

Note: The priorities presented above are informed by the companion paper, YAB consultation, and relevant literature.

## Conclusion

The Matilda Centre’s Youth Advisory Board rejected the idea of a “snowflake generation” outright. We view rising rates of mental ill-health not due to diminished resilience but more likely a product of increasingly hostile and unequal social conditions.

In Australia, young people face compounding stressors, including cost-of-living pressures, precarious employment, housing instability, and the long shadow of COVID-19. These concerns are not abstract headlines. They are the conditions of our daily realities, intensified by societal norms and social media algorithms that may proliferate and amplify trauma, and may encourage relentless comparison.

Reduced stigma around mental health has increased openness and help-seeking. However, *normalisation is not inflation.* We strongly caution against interpreting greater diagnostic visibility as overdiagnosis and suggest underdiagnoses amongst previous generations, particularly in Culturally and Linguistically Diverse and Aboriginal and Torres Strait Islander communities.

Overall, the YAB viewed the rise as real, meaningful, and systemic. Mental health symptoms are more common, more severe, and more functionally impairing. This is not just a cohort with better language for sadness, it is a generation navigating a system that is increasingly untenable and then being blamed for struggling within it.

We are not fragile. We are paying attention.

## Contributors

Georgie Berry conceived the article, conducted the literature review, interpreted the evidence, drafted the manuscript, revised the manuscript, and approved the final version for submission.

## Declaration of generative AI and AI-assisted technologies in the manuscript preparation process

During the preparation of this work, the author used Grammarly and ChatGPT for language editing. The author reviewed and edited the output as needed and takes full responsibility for the content of the published article.

## Declaration of interests

GB is supported by The Matilda Centre, The University of Sydney and reports receiving a grant from Children and Young People with Disability, as well as acting on the Youth Advisory Board for The Matilda Centre and the following volunteer roles; Board Director of Children and Young People with Disability Australia, National and Queensland Executive of EMILY’s List Australia, Branch Executive of the Australian Labor Party.
